# Outcomes following intradetrusor onabotulinumtoxinA in a national cohort of nursing home residents

**DOI:** 10.1002/bco2.472

**Published:** 2024-12-03

**Authors:** Leo D. Dreyfuss, Farnoosh Nik‐Ahd, Lufan Wang, Abigail Shatkin‐Margolis, Kenneth Covinsky, W. John Boscardin, Anne M. Suskind

**Affiliations:** ^1^ Department of Urology Weill Cornell Medical Center New York NY USA; ^2^ Department of Urology University of California, San Francisco San Francisco CA USA; ^3^ Department of Obstetrics and Gynecology University of California San Francisco CA USA; ^4^ Division of Geriatrics University of California San Francisco CA USA; ^5^ Department of Epidemiology and Biostatistics University of California San Francisco CA USA

**Keywords:** botox, elderly, frailty, nursing home, OnabotulinumtoxinA, overactive bladder

## Abstract

**Objectives:**

To determine predictors of treatment success and complications following intradetrusor onabotulinumtoxinA injections among a large cohort of nursing home (NH) residents, representing one of the most frail and vulnerable populations in the United States.

**Materials and methods:**

This is a retrospective cohort study of long‐stay NH residents who underwent onabotulinumtoxinA injections between 2014 and 2016. Residents were identified using the Minimum Data Set (MDS) linked to Medicare claims. Frailty was measured using the Claims‐based Frailty Index and socioeconomic status using the Area Deprivation Index (ADI; higher ADI = increasing social deprivation). The primary outcome was treatment success, defined as repeat onabotulinumtoxinA injection within 1 year of index injection. Secondary outcomes included 30‐day complications and urinary retention, defined as new indwelling urinary catheters identified on the MDS at 3 months.

**Results:**

OnabotulinumtoxinA injections were performed in 1683 NH residents. Mean age was 78.2 years, 74% were female and 22.8% had an indwelling urinary catheter at baseline. A total of 38.4% of residents had ≥1 30‐day complication and 14.6% had a new catheter at 3 months. Repeat injections were performed in 34.3% of residents within 1 year. Repeat injections were more likely among residents who were female [adjusted relative risk (aRR) 1.29; 95% CI 1.08–1.54] and who had a baseline catheter (aRR 1.30; 95% CI 1.11–1.52). Residents who were ≥85 years (aRR 0.78; 95% CI 0.64–0.96) and those in the lowest quartile ADI (aRR 0.75; 95% CI 0.61–0.93) were less likely to undergo repeat injections.

**Conclusion:**

Among this population of NH residents, who are by definition frail and comorbid, rates of repeat onabotulinumtoxinA injections are comparable to retrospective analyses of younger adults and independent of frailty and comorbidity. Based on these findings, surgeons should consider the entire clinical picture when evaluating patients for onabotulinumtoxinA injections and should not necessarily exclude those who are frail or comorbid from this potentially quality‐of‐life‐improving therapy.

## 1 INTRODUCTION

Overactive bladder (OAB) can be a debilitating condition that affects 16% of adults in the United States.^1^, ^2^ It also disproportionately affects older adults, increasing in incidence to over 30% for those over the age of 75.^3^ For older adults with OAB, intradetrusor onabotulinumtoxinA injection is an attractive treatment option with the potential to improve symptoms without the risks of systemic exposures to medical therapy.^4^, ^5^ These advantages have prompted clinicians to consider onabotulinumtoxinA injections as an option earlier in the OAB treatment pathway, particularly for older populations at risk for dementia associated with use of certain OAB medications.^6^

However, despite the burden of OAB in older adults, little is known about onabotulinumtoxinA injection use in this population. OAB research tends to focus on younger individuals and as a result older adults are underrepresented in the literature.^7^, ^8^, ^9^, ^10^, ^11^ Nursing home (NH) residents are a unique subset of older adults representing some of the most vulnerable individuals in the United States.^12^ This population tends to have high rates of frailty, which has been shown to independently predict adverse outcomes after surgery independent of comorbidity and age.^13^, ^14^, ^15^, ^16^ Given the concern for complications after treatment and a paucity of evidence supporting its use, clinicians may be hesitant to offer OnabotulinumtoxinA injections to NH residents, unnecessarily excluding a population from potentially life‐improving therapy.

Using the Minimum Data Set (MDS) linked to Medicare claims, this study seeks to identify efficacy and adverse events related to intradetrusor onabotulinumtoxinA injections for OAB in nursing home residents. The primary outcome was successful treatment with intradetrusor onabotulinumtoxinA injection, defined as repeat treatment within 1 year. Secondary outcomes include 30‐day complications and post‐procedure urinary catheter usage. Findings from this study will provide valuable insights into the management of OAB ‐ a highly burdensome problem ‐ in an extremely vulnerable population.

## 2 MATERIALS AND METHODS

### 2.1 Subjects and database

This study utilized the Minimum Data Set (MDS) 3.0 for Nursing Home Residents to identify long‐stay nursing home residents undergoing first‐time onabotulinumtoxinA injection from 2014 to 2016 and was deemed to be exempt by the institution's review board. The MDS is a mandatory assessment of all nursing home residents who reside in facilities that receive Medicare payments in the United States. It is obtained quarterly, with admission, readmission or with a change in clinical status. The MDS reports measures relating to cognitive, psychosocial and functional status, including information on urinary catheter use.^14^

Nursing home residents who underwent intradetrusor onabotulinumtoxinA injections during the study period were identified using the Current Procedural Terminology (CPT‐4) code 52287 from the Medicare Carrier claims. This CPT code is used to bill for chemodenervation procedures specific to the bladder and further methods to stratify residents according to indication (idiopathic, neuropathic) were not performed. To identify first‐time onabotulinumtoxinA injections, residents who underwent an onabotulinumtoxinA injection in the year prior to the index injection were excluded. For this analysis, residents were defined as long‐stay if they had at least 2 or more consecutive MDS assessments more than 30 days apart in the year prior to their index onabotulinumtoxinA injection procedure.

### 2.2 Covariates

Age, gender and other demographic data were obtained from the Medicare Master Beneficiary File from the year prior to the index onabotulinumtoxinA injection. Data for the Charlson Comorbidity Index (CCI) was abstracted from Medicare MedPar, Outpatient and Carrier files, using International Classification of Diseases (ICD) ‐9 and −10 codes as previously reported.^17^ The Area Deprivation Index (ADI) was used to measure socioeconomic status. The ADI is calculated using social and demographic factors associated with the beneficiary's 9‐digit zip code and has been shown to correlate with health outcomes. Zip codes with higher levels of social deprivation (lower socioeconomic status) are represented by higher ADI percentile levels.^18^

Frailty was measured using the Claims‐Based Frailty Index (CFI).^19^ The CFI is a weighted deficit accumulation model validated for use in Medicare data. It consists of 93 clinical variables [52 ICD‐9 and ‐10 codes, 25 CPT‐4 codes and 15 Healthcare Common Procedure Coding System (HCPCS) Level II Codes] and has been used across surgical disciplines to predict poor outcomes following invasive procedures.^13^, ^14^, ^15^, ^16^ The CFI was reported in tertiles, consistent with prior studies^20^: not frail to prefrail (CFI < 0.25), mildly frail (0.25 ≤ CFI < 0.35) and moderately to severely frail (CFI ≥ 0.35).

### 2.3 Outcome measures

The primary outcome was treatment success, defined by repeat onabotulinumtoxinA injections within one year of the index procedure, as it is assumed that residents who do not experience symptom improvement will not elect to undergo repeat injections. Due to high mortality rates among nursing home residents, repeat onabotulinumtoxinA injection procedure rates were calculated only among residents who were alive one year following the date of the index procedure.

Secondary outcomes included 30‐day post‐operative complications and the presence of a new urinary catheter within 3 months after the onabotulinumtoxinA injection. Thirty‐day complications were identified using ICD‐9/10 diagnosis codes, consistent with prior studies.^15^, ^16^, ^21^ New catheter utilization was determined by the MDS within 3 months of index procedure among residents without an indwelling urinary catheter at baseline or within the year prior to index procedure.^22^ Residents were considered to be catheter dependent if either the presence of an indwelling urethral catheter or intermittent catheterization was noted on the MDS assessment.

### 2.4 Statistical analysis

Categorical and continuous variables were described using a chi‐squared test and analysis of variance (ANOVA), respectively. Predictors of treatment success, represented by repeat onabotulinumtoxinA injections within 1 year of the index procedure, and the presence of a new urinary catheter within 3 months status post index procedure, were calculated using linear regression models with log link, Poisson distribution and robust standard errors. Independent variables in the model included age, race, sex, CCI, CFI, ADI and procedure calendar year. The model for repeat onabotulinumtoxinA injections also included the presence of a urinary catheter at baseline. If the number of outcome events was less than 10 per predictor variable, reduced models were constructed omitting predictors that were deemed not significant by the univariate model.

While the definition of repeat onabotulinumtoxinA injections was a repeat procedure performed within one year of the index injection, Kaplan–Meier analysis was performed for residents with more than one year of follow‐up available in the data to determine the cumulative incidence of repeat injections within 1, 2 and 3 years for those who had their index procedures performed in calendar years 2016, 2015 and 2014, respectively. Data management, statistical analyses and figure development were completed using SAS version 9.4. For all analyses, p < 0.05 was considered statistically significant. Per best‐practice guidelines for Medicare data, cell contents were masked if the number of events was less than 11.^23^

## 3 RESULTS

A total of 1683 long‐stay nursing home residents were identified who underwent intradetrusor onabotulinumtoxinA injections from 2014 to 2016. Table 1 reports the baseline characteristics of the study cohort. The average age of residents was 78.2 years and 26.0% were men. Mean CCI was 3.2 ± 2.6, 52.4% of residents were mildly frail (0.25 ≤ CFI < 0.35) and 26.9% of residents were severely frail (CFI ≥ 0.35). The presence of a baseline urinary catheter at was identified in 22.8% of residents.

**TABLE 1:** Baseline characteristics of long‐stay nursing home residents who underwent intradetrusor onabotulinumtoxinA injections from 2014 to 2016.

Variable	Total (N, %) N = 1683 (100.0%)
**Age in years**	
65–74	636 (37.8)
75–84	685 (40.7)
≥ 85	362 (21.5)
Mean ± SD	78.2 ± 7.5
**Sex**	
Men	438 (26.0)
Women	1245 (74.0)
**Race**	
White	1545 (91.8)
Non‐white	138 (8.2)
**Charlson Comorbidity Index**	
0–1	498 (29.59)
2–3	537 (31.91)
≥ 4	648 (38.50)
Mean ± SD	3.2 ± 2.6
**Claims‐based Frailty Index**	
Not Frail or Prefrail (CFI < 0.25)	350 (20.8)
Mildly Frail (0.25 ≤ CFI < 0.35)	881 (52.4)
Moderately to Severely Frail (CFI ≥ 0.35)	452 (26.9)
**Baseline catheter**	
Yes	384 (22.8)
**Area Deprivation Index National Quartile**	
Q1 (ADI 1–32)	432 (25.7)
Q2 (ADI 32–50)	462 (27.5)
Q3 (ADI 50–67)	438 (26.1)
Q4 (ADI ≥ 68)	347 (20.7)
**Calendar Year**	
2014	455 (27.0)
2015	591 (35.1)
2016	637 (37.9)

CFI=Claims‐based Frailty Index. ADI = Area Deprivation Index (higher ADI = lower socioeconomic status).

Overall, 38.4% of residents experienced at least one complication. The most common complication was urinary tract infection (UTI) in 28.6% of residents, followed by cardiovascular complications (10.5%) and pulmonary complications (6.0%). One‐year mortality was 10.5% among residents undergoing first‐time onabotulinumtoxinA injections (Table S1).

**FIGURE 1:**
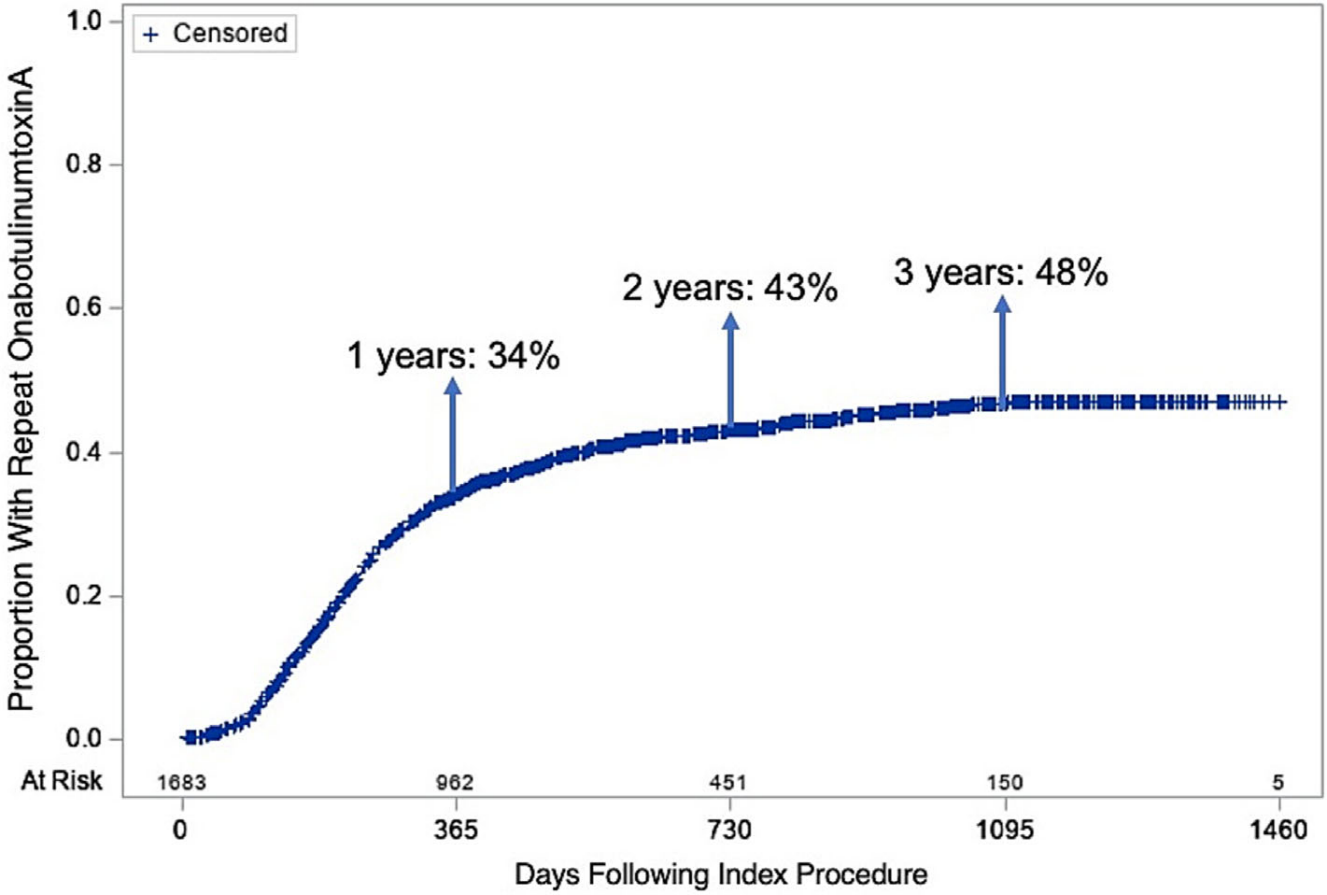
Cumulative incidence of repeat intradetrusor onabotulinumtoxinA injections up to 4 years following index procedure.

A total of 34.3% of residents had successful onabotulinumtoxinA injections, measured by the presence of a repeat injection within one year following the index procedure (Table 2). Female sex (adjusted relative risk (aRR) 1.29; 95% confidence interval (CI) 1.08–1.54) and presence of a urinary catheter at baseline (aRR 1.30, 95% CI 1.11–1.52) were associated with a higher relative risk of having repeat onabotulinumtoxinA injections at one year, compared to male residents and those without a baseline catheter, respectively. Compared to residents ages 64–74 years, residents aged ≥85 years (aRR 0.78; 95% CI 0.64–0.96) were less likely to undergo repeat injections within one year. Residents with higher ADI (lower socioeconomic status) were associated with a lower relative risk of undergoing repeat injections (aRR 0.81; 95% CI 0.67–0.98 for ADI Q3 and aRR 0.75; 95% CI 0.61–0.93 for ADI Q4, compared to ADI Q1). CFI and CCI were not associated with repeat onabotulinumtoxinA injections. Figure 1 illustrates the cumulative incidence of repeat injections up to 3 years following index onabotulinumtoxinA injections. For the subset of residents who had index onabotulinumtoxinA injections earlier in the study period and subsequently had longer follow‐up data available, rates of repeat procedures were 43% at 2 years and 48% at 3 years (Figure 1).

**TABLE 2:** Relative risk (RR) associated with undergoing repeat intradetrusor injection of onabotulinumtoxinA within 1 year of the initial procedure.

	Basic statistics			Univariate model RR		Multivariate model RR	
Variable name	Total, N (%) 1506 (100.0)	Event, n (%) 516 (34.3)	P value	Relative risk (RR, 95% CI)	Global P value	Relative risk (RR, 95% CI)	Global P value
**Age in years**							
65–74	583 (38.7)	224 (38.4)	0.015	Ref.	0.015	Ref.	0.037
75–84	610 (40.5)	200 (32.8)		0.85 (0.73–0.99)		0.87 (0.75–1.02)	
≥85	313 (20.8)	92 (29.4)		0.77 (0.63–0.93)		0.78 (0.64–0.96)	
**Race**							
White	1382 (91.8)	475 (34.4)	0.769	Ref.	0.767	Ref.	0.635
Non‐white	124 (8.2)	41 (33.1)		0.96 (0.74–1.25)		0.94 (0.73–1.22)	
**Gender**							
Men	366 (24.3)	105 (28.7)	0.010	Ref.	0.008	Ref.	0.003
Women	1140 (75.7)	411 (36.1)		1.26 (1.05–1.50)		1.29 (1.08–1.54)	
**Charlson Comorbidity Index**							
0–1	463 (30.7)	169 (36.5)	0.474	Ref.	0.4798	Ref.	0.713
2–3	488 (32.4)	163 (33.4)		0.92 (0.77–1.09)		0.93 (0.78–1.11)	
≥4	555 (36.9)	184 (33.2)		0.91 (0.77–1.07)		0.95 (0.79–1.15)	
**Claims‐based Frailty Index**							
Not Frail to Prefrail (CFI < 0.25)	329 (21.9)	126 (38.3)	0.216	Ref.	0.229	Ref.	0.262
Mildly Frail (0.25 ≥ CFI < 0.35)	785 (52.1)	259 (33.0)		0.86 (0.73–1.02)		0.87 (0.73–1.03)	
Moderately to Severely Frail (CFI ≥ 0.35)	392 (26.0)	131 (33.4)		0.87 (0.72–1.06)		0.87 (0.70–1.07)	
**Area Deprivation Index National Quartile**							
Q1 (ADI 1–32)	389 (25.9)	149 (38.3)	0.026	Ref.	0.025	Ref.	0.015
Q2 (ADI 32–50)	413 (27.5)	153 (37.1)		0.97 (0.81–1.16)		0.96 (0.81–1.15)	
Q3 (ADI 50–67)	386 (25.7)	121 (31.4)		0.82 (0.67–0.99)		0.81 (0.67–0.98)	
Q4 (ADI ≥ 68)	315 (21.0)	92 (29.2)		0.76 (0.62–0.94)		0.75 (0.61–0.93)	
**Baseline Catheter**							
No	1186 (78.8)	385 (32.5)	0.004	Ref.	0.005	Ref.	0.003
Yes	319 (21.2)	131 (41.1)		1.27 (1.08–1.48)		1.30 (1.11–1.52)	

CFI=Claims‐based Frailty Index. ADI = Area Deprivation Index (higher ADI = lower socioeconomic status). Model adjusted for procedure year. Observations ≤10 suppressed per Centers for Medicare & Medicaid Services (CMS) cell‐suppression policy.^23^

Among residents who did not have a urinary catheter at baseline, 14.6% had a new catheter within 3 months of their onabotulinumtoxinA injection. Table 3 shows the model depicting the relative risk (RR) of a new urinary catheter within 3 months following the index injection. Residents who were moderately to severely frail had a higher RR of having a new urinary catheter within 3 months, compared to residents who were not frail [aRR 1.39; 95% CI 0.68–2.85, p = 0.008]. Age, sex, race, CCI, CFI and ADI were not significantly associated with the presence of a new catheter use at 3 months. While the p‐value was <0.05 for CCI, the 95% CI coverage included 1.0 suggesting a lack of statistical significance. Following the creation of a reduced model with 12.8 events/predictor variable, CFI was no longer associated with new catheter use at 3 months (p = 0.613), suggesting the possibility of bias or over‐fitting of the uncondensed model (Table S2).

**TABLE 3:** Relative risk (RR) associated with new urinary catheter within 3 months following intradetrusor onabotulinumtoxinA injections. See Table S2 for the condensed model.

	Basic statistics			Univariate model RR		Multivariate model RR	
Variable name	Total, N (%) 439 (100.0)	Event, n (%) 64 (14.6)	P value	Relative risk (RR, 95% CI)	Global P value	Relative risk (RR, 95% CI)	Global P value
**Age in years**							
65–74	156 (35.5)	21 (13.5)	0.869	Ref.	0.868	Ref.	0.766
75–84	174 (39.6)	27 (15.5)		1.15 (0.68–1.95)		1.12 (0.66–1.91)	
≥85	109 (24.8)	16 (14.7)		1.09 (0.60–1.99)		1.24 (0.69–2.22)	
**Race**							
White	402 (91.6)	>53 (>13.2)	0.768	Ref.	0.780	Ref.	0.778
Non‐white	37 (8.4)	<11 (<29.7)		1.12 (0.52–2.43)		1.13 (0.50–2.58)	
**Gender**							
Men	104 (23.7)	20 (19.2)	0.124	Ref.	0.160	Ref.	0.057
Women	335 (76.3)	44 (13.1)		0.68 (0.42–1.10)		0.58 (0.35–0.95)	
**Charlson Comorbidity Index**							
0–1	143 (32.6)	16 (11.2)	0.058	Ref.	0.081	Ref.	0.189
2–3	141 (32.1)	17 (12.1)		1.08 (0.57–2.05)		0.95 (0.51–1.76)	
≥4	155 (35.3)	31 (20.0)		1.79 (1.02–3.13)		1.53 (0.86–2.74)	
**Claims‐based Frailty Index**							
Not Frail to Prefrail (CFI < 0.25)	73 (16.6)	11 (15.1)	0.005	Ref.	0.011	Ref.	0.012
Mildly Frail (0.25 ≥ CFI < 0.35)	238 (54.2)	24 (10.1)		0.67 (0.34–1.30)		0.66 (0.34–1.29)	
Moderately to Severely Frail (CFI ≥ 0.35)	128 (29.2)	29 (22.7)		1.50 (0.80–2.83)		1.53 (0.75–3.11)	
**Area Deprivation Index National Quartile**							
Q1 (ADI 1–32)	103 (23.5)	>15 (>14.6)	0.097	Ref.	0.093	Ref.	0.108
Q2 (ADI 32–50)	111 (25.3)	<11 (<9.9)		0.58 (0.28–1.22)		0.56 (0.27–1.16)	
Q3 (ADI 50–67)	117 (26.7)	24 (20.5)		1.32 (0.74–2.35)		1.25 (0.69–2.25)	
Q4 (ADI ≥ 68)	107 (24.4)	14 (13.1)		0.84 (0.43–1.64)		0.86 (0.45–1.65)	

CFI=Claims‐based Frailty Index. ADI = Area Deprivation Index (higher ADI = lower socioeconomic status). Model adjusted for procedure year. Observations ≤10 suppressed per Centers for Medicare & Medicaid Services (CMS) cell‐suppression policy.^23^

## 4 DISCUSSION

This study represents the largest series of nursing home residents who underwent intradetrusor onabotulinumtoxinA injections for the treatment of OAB in the literature. Despite this population having a mean age of 78 years and 70% being mildly‐severely frail, 34% had a repeat onabotulinumtoxinA injection procedure in 1 year independent of age and only 14.6% required a new urinary catheter. For residents with available longer follow‐up, rates of repeat procedures increased to 48% by 3 years. These findings were independent of age, frailty and comorbidity, and are comparable to other retrospective studies in the literature on younger, community‐dwelling populations.^9^, ^24^, ^25^

Little is known about the long‐term success of onabotulinumtoxinA, particularly in older populations. Prospective trials indicate that up to 60% of individuals in the general population will experience significant short‐term improvement in OAB symptoms following intradetrusor onabotulinumtoxinA injections.^7^, ^9^ Long‐term success rates measured by the proportion of subjects undergoing repeat onabotulinumtoxinA injections are limited to smaller cohorts, with rates of repeat procedure ranging from 53 to 58%^25^, ^26^ at a median time of 377–475 days between first and second injections. In the present analysis, the 1‐year rate of repeat onabotulinumtoxinA injection was considerably lower at 34%, however, this rate increased to 48% at 3 years among beneficiaries with longer follow‐up. Furthermore, these findings are similar to a previous claim‐based study using New York residents with a mean age of 61.7 years, in which 28% and 41.2% of subjects received a second injection within 1 and 3 years, respectively.^24^ While the reasons for this difference are unclear and may be related to differences in baseline characteristics between cohorts, findings from this study may offer some insight into the potential *real world* success rates of onabotulinumtoxinA injections, as measured by repeat injections, in the vulnerable population of nursing home residents as well as older adults overall.

Younger age, female sex, higher socioeconomic status and the presence of a urinary catheter at baseline were all independently associated with higher rates of repeat onabotulinumtoxinA injections, whereas comorbidity and frailty were not. The impact of age on improved patient‐reported outcomes following onabotulinumtoxinA injections for OAB has been evaluated in several series. For example, two secondary analyses of the ROSETTA trial – a multicenter randomized trial comparing onabotulinumtoxinA injection to sacral neuromodulation among women with refractory OAB – demonstrated that older subjects were less likely to report symptom improvement following onabotulinumtoxinA injection.^27^, ^28^ Frailty was not assessed in this cohort. Another retrospective cohort of 166 subjects demonstrated that older adults had lower rates of subjective symptom improvement at 12 months independent of frailty.^10^ Interestingly, frailty and comorbidity were not associated with repeat onabotulinumtoxinA injections in the present analysis. Residents with higher ADI (lower socioeconomic status) were less likely to undergo repeat onabotulinumtoxinA injection independent of race. The ADI has been previously shown to correlate with decreased healthcare utilization and is associated with poverty, unemployment and diminished access to transportation healthcare resources.^18^ These findings suggest social factors – in addition to clinical characteristics – likely play a role in adherence to OAB therapy among nursing home residents and warrants further investigation.

Thirty‐day Complications were observed in 38.4% of residents in the present analysis, 74.5% of which were UTIs. While complications reported herein cannot necessarily be attributed directly to onabotulinumtoxinA injections and may represent incidental events unrelated to the index procedure, this rate is comparable to prior rates reported in the literature. In one retrospective cohort of 217 subjects, 44.7% of whom were 75 years or older, 52.1% experienced adverse events with UTI being the most common (22% of women and 7% of men).^9^ Similarly, UTI rates were reported to be 31% for women less than 65 years and 43% for women older than 65 years in a secondary analysis of the ROSETTA trial.^28^ While this rate of UTI's is high, it is also possible that a proportion was unrelated to the index procedure, as supported by previously reported UTI rates among nursing home residents being as high as 21.8%.^29^

Urinary retention is a well‐described risk associated with onabotulinumtoxinA injections.^7^, ^9^ The present analysis measured urinary retention by the presence of a urinary catheter within 3 months following the index procedure. Overall, 14.6% of residents required new urinary catheter use in this study. These findings are comparable to rates of urinary retention seen by Komesu et al (22.8% for <65 years, 17.6% for ≥65 years, OR),^28^ although reported rates of urinary retention after onabotulinumtoxinA injection are variable and likely related to treatment dose,^7^, ^9^, ^10^ which is not available in these data. These findings will allow clinicians to better counsel patients on risks prior to invasive OAB therapy and should not preclude treatment of nursing home residents – who may have limited mobility and poor social support – with onabotulinumtoxinA injection.

Results from this study must be considered in the context of a few limitations. While this cohort of nursing home residents represents a unique population with high rates of frailty and comorbidity, nursing home residents are a particularly vulnerable population with 1‐year mortality rates higher than the general population.^12^ As such, extrapolation of results to populations of community‐dwelling older adults should be undertaken with caution. Utilization of Medicare data provides a large, highly generalizable sample size but lacks subjective patient‐reported outcomes regarding symptom improvement. Repeat onabotulinumtoxinA injection was used as a surrogate for treatment success, consistent with prior studies,^24^ yet this definition of success differs from prior landmark trials using patient‐reported outcomes^7^ and may underreport success as some residents may have maintained symptom resolution longer than 1 year. However, any reason for the abandonment of treatment or lack of repeat treatment within one year – including inadequate symptom relief, adverse events or logistical issues related to the cessation of treatment – can be considered an “unsuccessful” treatment and is reflected in the primary outcome. Complications were measured within 30 days of the index procedure, but causality is uncertain. This method is a standard among published studies in the literature^14^, ^15^, ^16^, ^21^ and, while direct attribution of specific complications to onabotulinumtoxinA injection versus other causes (anaesthesia‐related, e.g.) cannot be made, there is a high likelihood that these complications are related to the index procedure. The CFI has been shown to predict poor surgical outcomes in Medicare beneficiaries,^16^ but its utility in a population of nursing in nursing home residents – the majority of whom are frail – is less clearly understood. Multivariable logistic regression was performed to account for confounding, but it is possible that unmeasured variables may influence findings. Finally, claims‐based analyses are limited by potential errors in billing codes which can influence results.

Despite these limitations, this study presents important findings from the largest reported cohort of nursing home residents, an under‐studied and vulnerable population with many unique challenges associated with the management of OAB. As the population in the United States continues to age and third‐line therapies for OAB are pursued earlier in the treatment pathway, results such as these may encourage providers to utilize onabotulinumtoxinA injection when managing nursing home residents, despite high rates of comorbidity and frailty.

## 5 CONCLUSION

This analysis represents the largest series of nursing home residents undergoing intradetrusor onabotulinumtoxinA injection and shows that 34% will undergo repeat treatment in 1‐year independent of comorbidity or frailty with complication rates similar to that in younger cohorts. Nearly 50% of those with longer follow‐ups will undergo repeat treatment for 3 years. These findings suggest that intradetrusor onabotulinumtoxinA injection may be an appropriate option for select nursing home residents with OAB.

## CONFLICT OF INTEREST STATEMENT

The authors have declared that no conflict of interest exist**.**

## Supporting information


**Table S1:** Thirty‐day complications and 1‐year mortality following intradetrusor onabotulinumtoxinA injections among nursing home residents.
Table S2: Condensed model showing relative risk (RR) associated with new urinary catheter within 3 months following intradetrusor onabotulinumtoxinA injections.
