# Supplementary material for: Outcomes following intradetrusor onabotulinumtoxinA in a national cohort of nursing home residents
Source: BJUI Compass. 2024 Dec 3;6(1):e472. doi: 10.1002/bco2.472 (PMC11771503; doi:10.1002/bco2.472)
Supplement: Supplementary file 1 — Table S1: Thirty‐day complications and 1‐year mortality following intradetrusor onabotulinumtoxinA injections among nursing home residents. Table S2: Condensed model showing relative risk (RR) associated with new urinary catheter within 3 months following intradetrusor onabotulinumtoxinA injections. [file BCO2-6-e472-s001.docx]

Supplemental Table 1: Thirty-day complications and 1-year mortality following intradetrusor onabotulinumtoxinA injections among nursing home residents.

| **Complication** | **Total, N (%)**  **1683 (100.0)** |
| --- | --- |
| Complication ≥ 1 | 642 (38.4) |
| **Complication type** |  |
| UTI | 478 (28.6) |
| Cardiovascular | 175 (10.5) |
| Pulmonary | 101 (6.0) |
| Acute renal failure | 56 (3.4) |
| DVT/PE | 46 (2.8) |
| Other infection | 31 (1.9) |
| Delirium | 12 (0.7) |
| Wound complication | <11 (<1.0) |
| Reoperation | <11 (<1.0) |
| Postoperative hemorrhage | <11 (<1.0) |
| Postoperative stroke | <11 (<1.0) |
| Other complications | <11 (<1.0) |
| Postoperative shock | <11 (<1.0) |
| **1-Year mortality** | 177 (10.5) |

*UTI=Urinary Tract Infection, DVT/PE=Deep Vein Thrombosis/Pulmonary Embolus. Observations ≤10 suppressed per Centers for Medicare & Medicaid Services (CMS) cell-suppression policy (23).*

Supplemental Table 2: Condensed model showing relative risk (RR) associated with new urinary catheter within 3 months following intradetrusor onabotulinumtoxinA injections.

|  | **Basic Statistics** | | | **Univariate Model RR** | | **Multivariate Model RR** | |
| --- | --- | --- | --- | --- | --- | --- | --- |
| **Variable Name** | **Total, N (%)**  439 (100.0) | **Event, n (%)** 64 (14.6) | **P value** | **Relative risk (RR, 95% CI)** | **Global P value** | **Relative risk (RR, 95% CI)** | **Global P value** |
| **Gender** |  |  |  |  |  |  |  |
| Men | 104 (23.7) | 20 (19.2) | 0.124 | Ref. | 0.157 | Ref. | 0.180 |
| Women | 335 (76.3) | 44 (13.1) |  | 0.68 (0.42 - 1.10) |  | 0.70 (0.44 - 1.13) |  |
| **Charlson Comorbidity Index** |  |  |  |  |  |  |  |
| 0 | 143 (32.6) | 16 (11.2) | 0.058 | Ref. | 0.081 | Ref. | 0.068 |
| 1 - 3 | 141 (32.1) | 17 (12.1) |  | 1.08 (0.57 - 2.05) |  | 1.11 (0.59 - 2.07) |  |
| ≥4 | 155 (35.3) | 31 (20.0) |  | 1.79 (1.02 - 3.13) |  | 1.87 (1.07 - 3.25) |  |
| **Claims-based Frailty Index** |  |  |  |  |  |  |  |
| Not Frail to Prefrail (CFI<0.25) | 73 (16.6) | 11 (15.1) | 0.897 | Ref. | 0.898 | Ref. | 0.613 |
| Mildly to Severely Frail (CFI≥0.25) | 366 (83.4) | 53 (14.5) |  | 0.96 (0.53 - 1.75) |  | 0.84 (0.44 - 1.59) |  |
| **Area Deprivation Index National Quartile** |  |  |  |  |  |  |  |
| ADI 1 - 50 | 214 (48.9) | 26 (12.2) | 0.154 | Ref. | 0.153 | Ref. | 0.205 |
| ADI 51 - 100 | 224 (51.1) | 38 (17.0) |  | 1.40 (0.88 - 2.22) |  | 1.35 (0.84 - 2.18) |  |

*CFI=Claims-based Frailty Index. ADI=Area Deprivation Index (higher ADI=lower socioeconomic status). Model adjusted for Age, Race, and procedure year.*
